# A deep study of the protection of Lithium Cobalt Oxide with polymer surface modification at 4.5 V high voltage

**DOI:** 10.1038/s41598-018-19176-6

**Published:** 2018-01-16

**Authors:** ZhiXiong Yang, RenGui Li, ZhengHua Deng

**Affiliations:** 1 Chengdu Institute of Organic Chemistry, the Chinese Academy of Sciences, Chengdu, 610041 China; 2 University of Chinese Academy of Sciences, Beijing, 100039 China

## Abstract

Charging the cells above a conventional voltage of 4.2 V is a promising attempt to increase the energy density of Lithium Cobalt Oxide (LCO), however, the problem of crystal instability at high voltage that leading deterioration of cycle performance needs to be urgently resolved. In this work, as an effective and easy approach to improve the cycle performance and crystal stability of LCO cycling at 4.5 V high voltage, we demonstrate direct surface modification of a LCO cathode by poly [N,N-bis(2-cryano-ethyl)-acrylamide]. The results of SEM, TEM and XRD all indicate that the crystal structure of polymer coating LCO remains unchanged after cycling at 4.5 V high voltage for 60 times. Furthermore, the XPS study of valence of cobalt on the surface of LCO demonstrates that cobaltic ion of polymer coating LCO can be reduced to cobaltous ion after charging the cell. Thus, the activity of the crystal surface can be weakened, as a result, the stability is improved, leading to the performance improvement.

## Introduction

Lithium-ion batteries are small in size, light in weight and high in energy density, so applications of lithium-ion batteries are expanding into new fields such as smart mobile devices, electric vehicles and energy storage systems^1–3^. Among various cathode materials of lithium-ion batteries, LiCoO_2_ is very popular in commercialized batteries. Although the theoretic capacity of LiCoO_2_ is 273 ${\rm{m}}{\rm{A}}{\rm{h}}\,{{\rm{g}}}^{-1}$, limited by its crystal structure, the capacity of commercialized LiCoO_2_ battery is only about 140–150 ${\rm{m}}{\rm{A}}{\rm{h}}\,{{\rm{g}}}^{-1}$. The capacity of LiCoO_2_ depends on the amount of ${{\rm{Li}}}^{+}$ that the crystal releases by charge, the amount of released ${{\rm{Li}}}^{+}$ depends on the charge cut-off voltage, the higher the charge voltage is, the more ${{\rm{Li}}}^{+}$ the crystal losses. When the cut-off voltage is increased to 4.5 V, the capacity is approximately 220 ${\rm{m}}{\rm{A}}{\rm{h}}\,{{\rm{g}}}^{-1}$ ^4^. But with the crystal losing more **Li**^**+**^, the crystal structure becomes unstable, at the same time, unwanted interfacial side reactions between charged LiCoO_2_ and liquid electrolytes leads to cobalt loss and electrolyte decomposition^4–6^. As a result, the deterioration of cell performance happens.

As an effective approach to overcome these drawbacks of LiCoO_2_ at high voltage, the surface modification of LiCoO_2_ with polymer materials has been extensively investigated. Polymer materials have advantages of coating uniformity, flexibility and ease of preparation. Sang-sung Lee *et al*. have reported that LiCoO_2_ coated with polyimide gel polymer electrolyte shows a good performance at 4.4 V cut-off voltage. Its initial capacity reaches 160 ${\rm{m}}{\rm{A}}{\rm{h}}\,{{\rm{g}}}^{-1}$, and still remains 120 ${\rm{m}}{\rm{A}}{\rm{h}}\,{{\rm{g}}}^{-1}$ after 100 cycles^7^. Then they also used poly (ethylene glycol diacrylate) gel polymer electrolyte to coat LiCoO_2_^8^. Poly pyrrole^9^ and C60^10^ are also reported to coat LiCoO_2_.

We believe that the problems related to the charged LiCoO_2_ at high voltage are mainly due to the oxidation of cobalt ion on the surface^11,12^. If the activity of cobalt ions on the surface of the charged LiCoO_2_ could be reduced, the performance of high voltage LiCoO_2_ cell would be improved significantly. In this study, a new monomer N, N-bis (2-cryano-ethyl)-acrylamide) (BCEAM)^13^ was synthesized(supporting information Figure S1). The isolated electron pair at N atom in nitrile group of BCEAM can interact with the cobalt ions on the LiCoO_2_ surface, and change the chemical environment of it. At the same time, taking the conformation into account, the nitrile contacts with the amido though two methylenes, which provides a better flexibility to combine with cobalt ions. The surface modified LiCoO_2_(MLCO) coating with poly [N,N-bis(2-cryano-ethyl)-acrylamide]) (PBCEAM) was prepared by *in situ* polymerization. By this way, compared to the pristine LiCoO_2_ (PLCO), the cycle performance of high voltage (4.5 V) LiCoO_2_ cell is improved significantly. SEM, XRD and TEM are used as preferred techniques for the characterization of the surface morphology and crystal structure of LiCoO_2_. XPS is chosen to examine the valence of cobalt on the surface of LiCoO_2_. Based on these results, the protection mechanism of LiCoO_2_ cycling at high voltage is discussed.

## Results and Discussion

At first, we should confirm that the monomer had been adsorbed on the surface of LiCoO_2_ and turned to polymer. Table 1 lists the atomic percentage of elements on the surface of PLCO and MLCO, which is based on the results of energy dispersive X-ray analysis (supporting information Figure S2). Because there is carbon in the conductive adhesive for SEM sample preparation, carbon can still be detected for PLCO, the atomic ratio of carbon to cobalt of PLCO is 0.108. However, the atomic ratio of carbon to cobalt of MLCO is 0.233, which is more than two times of that of PLCO. Second, the atomic ratio of oxygen to cobalt of MLCO (1.691) is higher than that of PLCO (1.451). Third, the nitrogen exists only in MLCO. These results indicate there is an organic material on the surface of MLCO.

**Table 1:** The Atomic percentage of elements on the surface of PLCO and MLCO.

Material	Atomic percentage of elements [%]				Atomic Ratio	
	C	N	O	Co	C/Co	O/Co
PLCO	4.22	—	56.7	39.08	0.108	1.451
MLCO	7.87	1.2	57.14	33.79	0.233	1.691

**Figure 1:**
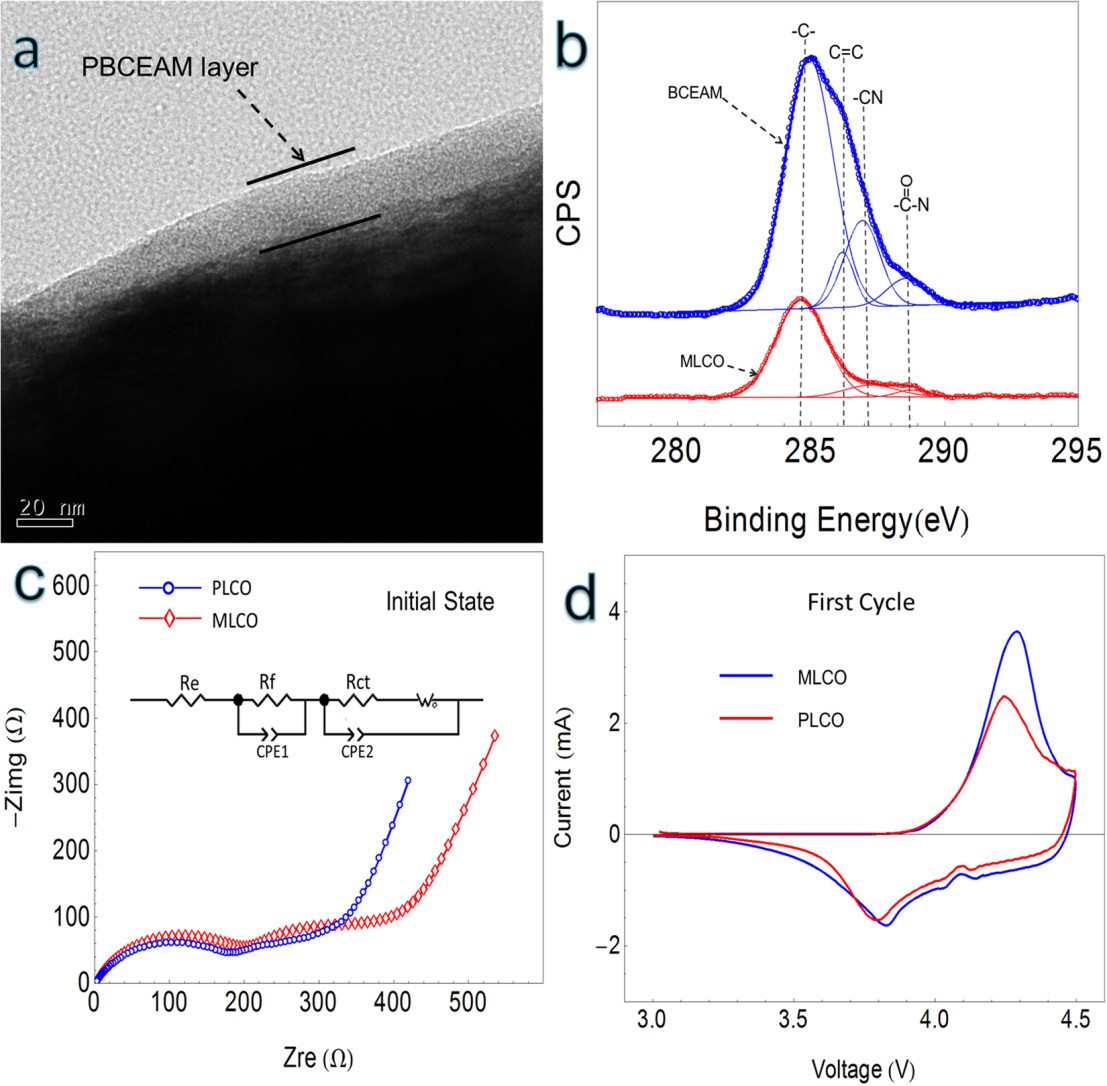
Polymer-wrapping structrue. (**a**) The TEM image of MLCO. (**b**) The C1s XPS spectrums of BCEAM and MLCO. (**c**) The Electrochemical Impedance Spectroscopy (EIS) of the cells of MLCO and PLCO. (**d**) The Cyclic Voltammograms (CV) of the cells of MLCO and PLCO.

Figure 1 shows the structure analyses of MLCO. The successful encapsulation of LiCoO_2_ with the PBCEAM layer is displayed in Fig. 1a. On the surface of MLCO, the PBCEAM forms a smooth coating layer with a thickness of around 20 nm. Figure 1b shows the C1s XPS spectrums of BCEAM and MLCO. In the Figure, four kinds of chemical carbon bonds can be distinguished in the spectrum of BCEAM, there are a carbon-carbon single bond, a carbon-carbon double bond, a carbon-nitrogen triple bond and a carbon-oxygen double bond. In the spectrum of MLCO, there are three kinds of chemical carbon bonds can be distinguished, there are a carbon-carbon single bond, a carbon-nitrogen triple bond and a carbon-oxygen double bond, except for the carbon-carbon double bond. This result indicates that the C=C bonds of monomer adsorbed on the surface of MLCO has turned to C–C single bonds of polymer, namely, a PBCEAM modification layer can be built on the surface of MLCO by *in situ* polymerization. Thus, the PBCEAM has uniformly coatinged on the surface of the MLCO.

Figure 1c shows the Electrochemical Impedance Spectroscopy (EIS) of the cells of PLCO and MLCO. According to the fit results of the Nyquist plots, the impedance of the passivation interface layers (${R}_{f}$) of MLCO (220 Ω) are a little higher than that of the PLCO (204 Ω), the charge transfer resistance (${R}_{{ct}}$) of MLCO (88 Ω) are higher than that of PLCO (25 Ω), these may due to the insulation of the polymer layer on the surface. However, this acceptable increase of the impedance doesn’t obviously obstacle the transportation of ${{\rm{Li}}}^{+}$ during the electrochemical reactions. As Fig. 1d shows, the Cyclic Voltammograms (CV) of the MLCO and PLCO at first cycle are very similar, the MLCO has the oxidation and reduction potentials that very close to those of PLCO.

**Figure 2:**
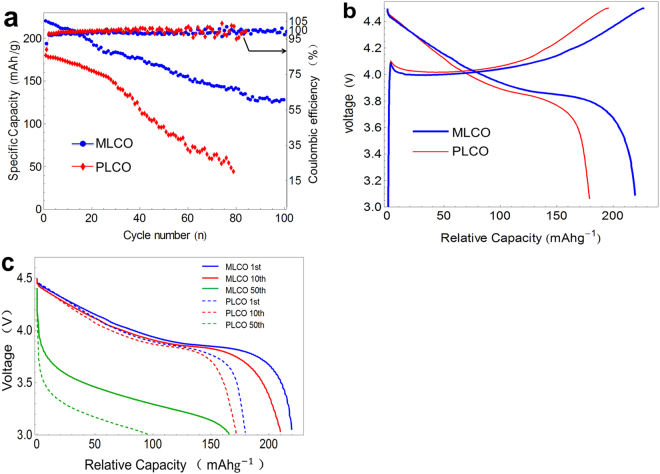
Cyclability. (**a**) the cycle performance of PLCO and MLCO. (**b**) The first charge and discharge profiles of PLCO and MLCO. (**c**) discharge profiles of PLCO and MLCO of different cycles.

Figure 2 displays the effect of PBCEAM modification layer on the cycle and discharge performance of cells charged to 4.5 V at a current density of 0.2 C. As Fig. 2a shows, the MLCO exhibits a significant improvement of cycle property compared to PLCO. The first discharge capacity of MLCO is as much as 220 ${\rm{m}}{\rm{A}}{\rm{h}}\,{{\rm{g}}}^{-1}$, and still remains 120 ${\rm{m}}{\rm{A}}{\rm{h}}\,{{\rm{g}}}^{-1}$ after 100 cycles. However, the first capacity of PLCO is just about 180 ${\rm{m}}{\rm{A}}{\rm{h}}\,{{\rm{g}}}^{-1}$, which is much lower than MLCO, and only 44 ${\rm{m}}{\rm{A}}{\rm{h}}\,{{\rm{g}}}^{-1}$ left at 80th cycle. Besides, after 30 cycles, the capacity fading of the PLCO accelerates with increasing cycle number, whereas the capacity fading of MLCO is retarded. Figure 2b shows the first charge and discharge profiles of PLCO and MLCO cells charged to 4.5 V at a current density of 0.2 C. Both the MLCO and PLCO show a conventional voltage plateau, but the charge plateau of MLCO is lower than PLCO, the discharge plateau of MLCO is higher PLCO. The MLCO also has a higher coulombic efficiency than that of PLCO, the coulombic efficiency of MLCO and PLCO are 95.6% and 92.3% respectively. This results may due to the side reaction retardant of the polymer modification layer on the MLCO, which makes its’ capacity and coulombic efficiency higher than those of PLCO.

Not only the differences of discharge performance for the first cycle, but also the differences of discharge performance for more cycles can better demonstrate the effect of the polymer modification. As Fig. 2c shows, at first 10 cycles, both MLCO and PLCO show a good discharge performance, but after more cycles, especially for 50^th^ cycle, the polarization of PLCO is so serious that its’ discharge plateau has shifted to a very low voltage, and its discharge capacity is less than 100 ${\rm{m}}{\rm{A}}{\rm{h}}\,{{\rm{g}}}^{-1}$. However, with the protection of the polymer modification layer, a discharge plateau of MLCO that much higher than PLCO is observed, and the discharge capacity still remains 166 ${\rm{m}}{\rm{A}}{\rm{h}}\,{{\rm{g}}}^{-1}$. As to the increasing polarization of MCLO cells that still exists during cycling, this can be explained by considering the lithium dendrite of the lithium metal counter electrode^14–16^ and the decomposition of electrolyte at high potential of 4.5V^17^. The cycling voltammetry (CV) of the electrolyte is given in supporting information Fig. S3, there is an oxidation peak for the electrolyte between 4 V and 5 V. Based on these two main reasons, the cycle property of MLCO cell is affected negatively. As to PLCO, except for these drawbacks, the instability of its crystal cycling at high voltage affects its cycling stability worse. And this will be discussed in the following part.

**Figure 3:**
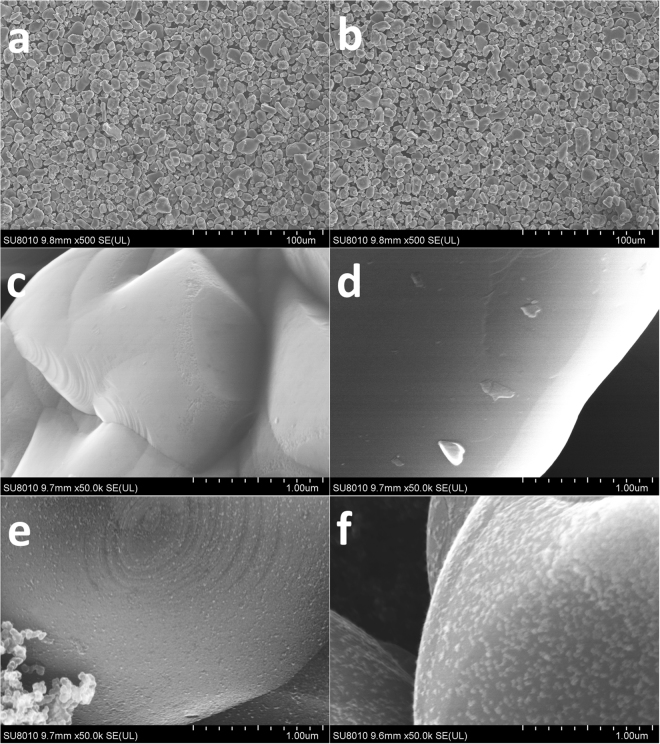
Surface morphology. The SEM images of: (**a**) and (**c**) MLCO; (**b**) and (**d**) PLCO; (**e**) MLCO after 60 cycles; and (**f**) PLCO after 60 cycles.

Figure 3 displays the SEM images of MLCO (**a** and **c**), PLCO (**b** and **d**), MLCO after 60 cycles (**e**) and PLCO after 60 cycles (**f**). Before cycling, the powder structure and surface morphology of PLCO and MLCO are very similar, they are very smooth and even some textures can be seen. But after cycling for 60 times, their surface became very different. For MLCO, although there are some sediment particles on the surface, the ring texture is still clearly visible. However, for PLCO, the surface became much coarser, and the bigger bright spots on the surface are obviously not the sediments, this may due to the phase transformation of the crystal, which will be discussed in the following part. This difference indicates that the coating polymer provides a good protection for LiCoO_2_ surface.

**Figure 4:**
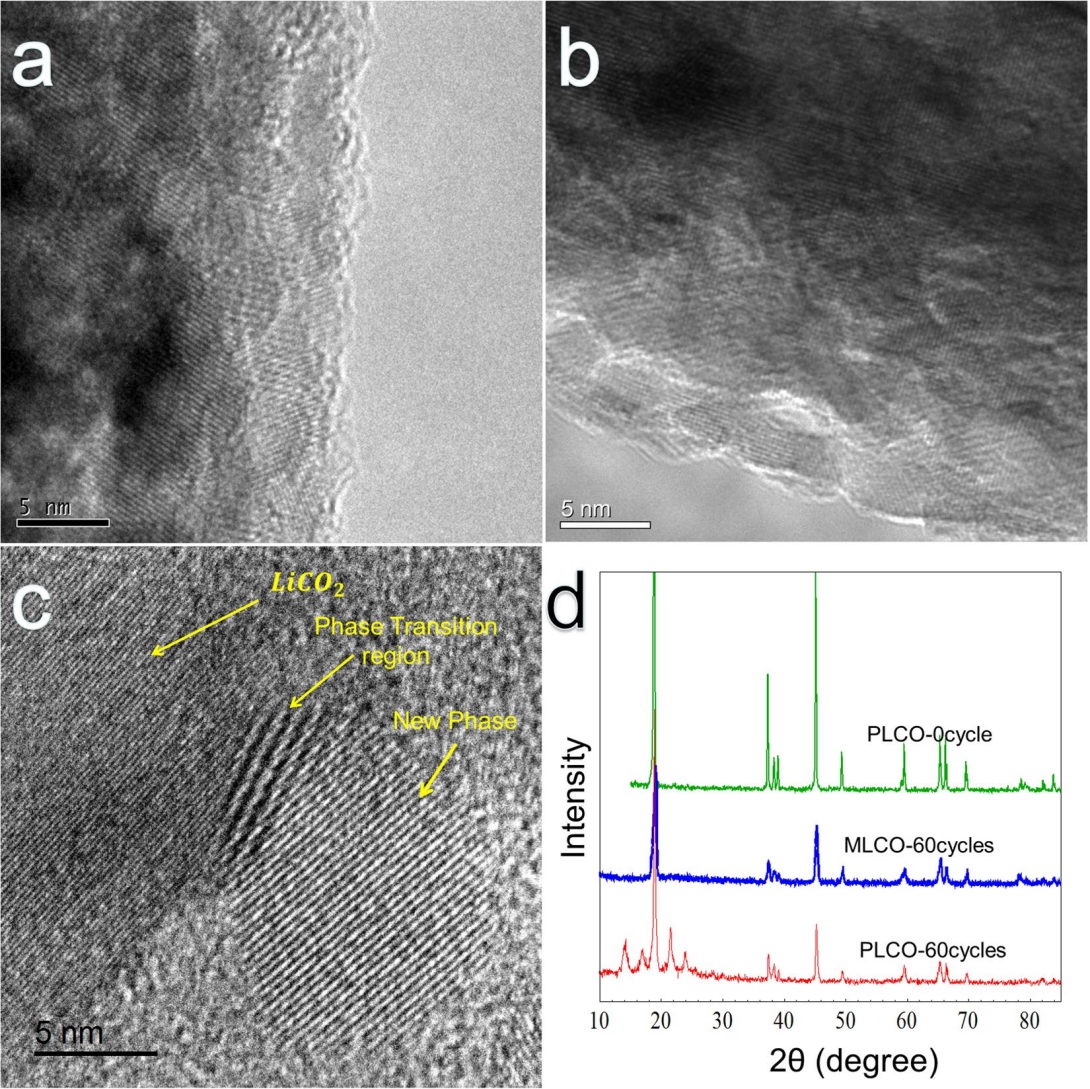
LiCoO_2_ crystal structure after 60 cycles. (**a**) The HRTEM image of MLCO. (**b**) The HRTEM image of MLCO after 60 cycles. (**c**) The HRTEM image of PLCO after 60 cycles. (**d**) The XRD patterns of PLCO powders (PLCO-0 cycle), cathode of MLCO (MLCO-60 cycles) and cathode of PLCO (PLCO-60 cycles) after 60 cycles.

Figure 4 shows the crystal structure change of MLCO and PLCO after 60 cycles. As Fig. 4b shows, for the MLCO, the lamellar structure of LiCoO_2_ crystal saved perfectly after 60 cycles^18^, which is the same with that of the MLCO at initial state (Fig. 4a). However, as Fig. 4c shows, after 60 cycles, the PLCO undergoes a phase transformation: the lattice planes of the crystal bend to form a phase transition regeion^5,18^, then these lattice planes turn to a new crystall that totally different from LiCO_2_ with a widder interplaner distance. This phase transformation leads to the surface morphology change of PLCO that observed in the SEM images (Fig. 3d). This result can also be proved by XRD. Figure 4d shows the XRD patterns of PLCO powders (PLCO-0 cycle), electrode of PLCO (PLCO-60 cycles) and MLCO (MLCO-60 cycles) after 60 cycles. The patterns of PLCO and MLCO electrode cycled for 60 times are the same. However, the pattern of PLCO electrode after 60 cycles has 4 new peaks in the 2 theta range from 10° to 25°, except for the main peak at about 19°, which is corresponding to the new crystal observed by HRTEM (Fig. 4c). These results prove that the crystal of MLCO can be protected from transformation with the polymer modification.

Furthermore, in order to figure out the protection mechanism of MLCO, the XPS was utilized to examine the valence of cobalt on the surface of PLCO and MLCO before and after charge.

**Figure 5:**
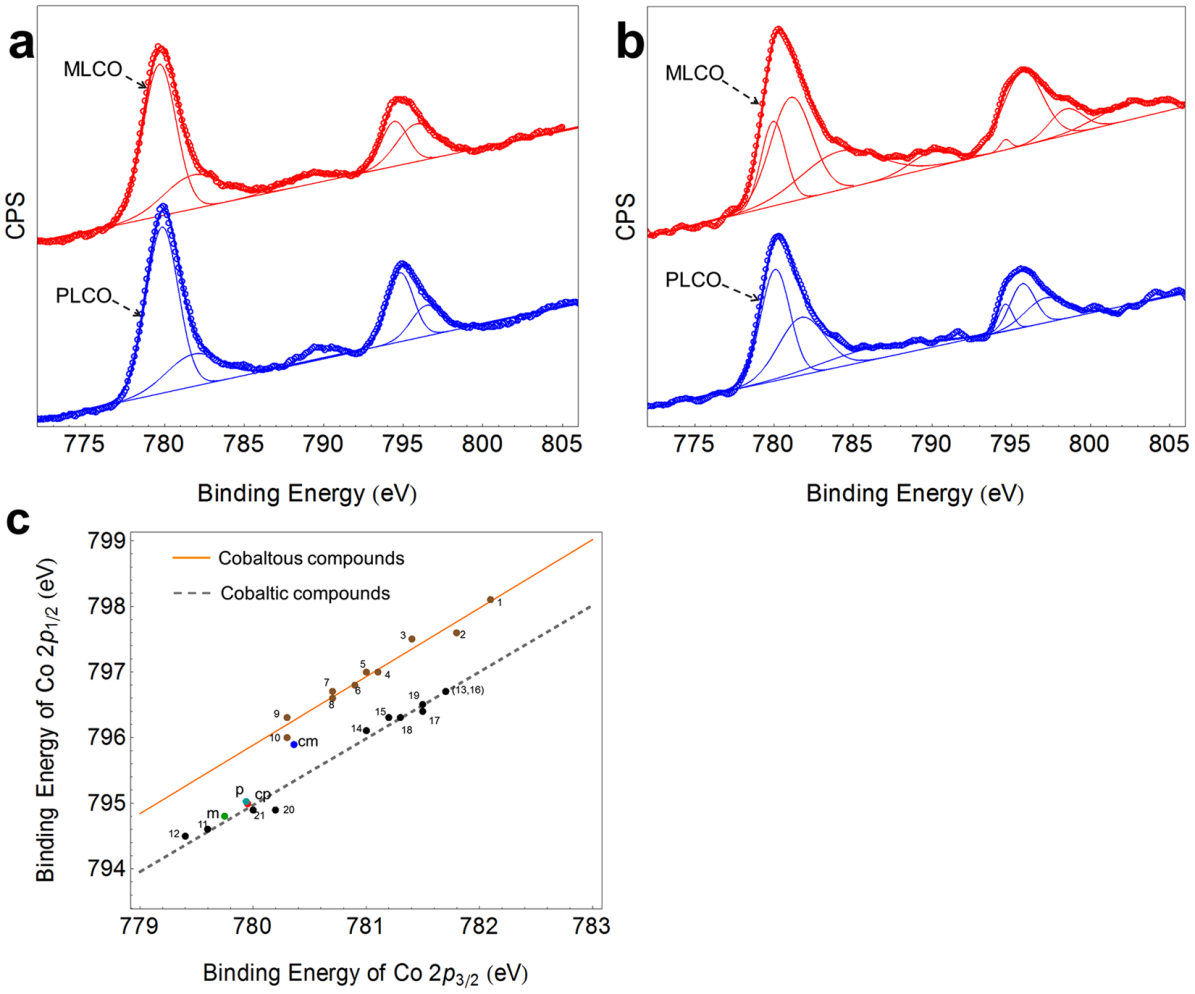
The XPS characterization of MLCO and PLCO: the XPS spectrums of Co at 2p level for PLCO and MLCO before (**a**) and after (**b**) charge. The co-relations of the binding energies of ${{\rm{C}}{\rm{o}}2{\rm{p}}}_{3/2}$ and ${{\rm{C}}{\rm{o}}2{\rm{p}}}_{1/2}$ of cobalt compounds (**c**), see supporting information Table S1 for a listing of the compounds. The point p (cyan), cp (red), m (green), cm (blue) are corresponded with PLCO, PLCO in charge state, MLCO, and MLCO in charge state, respectively.

From the XPS measurements, Cobaltous compounds cannot be distinguished from cobaltic compounds by chemical shift alone, but they can be discriminated by the satellite structure. Besides, the spin-orbit splittings of 2p level (ΔE, the energy difference between ${{\rm{C}}{\rm{o}}2{\rm{p}}}_{3/2}$ and ${{\rm{Co}}2{\rm{p}}}_{1/2}$) is also very useful to distinguish the valence states of cobalt compounds, because it is clear that for cobaltic compounds, the ΔE is 15.0 eV and for cobaltous compounds, the ΔE is 16.0 eV^19^. And, there is also an interesting relation between the binding energies of ${{\rm{C}}{\rm{o}}2{\rm{p}}}_{3/2}$ subshells and ${{\rm{Co}}2{\rm{p}}}_{1/2}$ subshells, it is that two different linear co-relations are easily obtained for the cobaltic and cobaltous compounds, which is shown in Fig. 5c^19^.

Figure 5 displays the XPS spectrums of Co at 2p level for PLCO and MLCO before (a) and after (b) charge, the spectrums for charged PLCO and MLCO were examined where the cells were charged to 4.5 V at a current density of 0.2 C and disassembled in a dry room to remove the charged cathode. In the initial state, as Fig. 5a shows, the satellite type of PLCO and MLCO are the same, but the positions and the band widths at half height of the main peaks are different. Although the chemical shift cannot distinguish the oxide states of cobalt compounds, it reflects the over-all electron density. Table 2 lists the chemical shifts after the pristine LiCoO_2_ is modified with PBCEAM, they are −0.2 eV for ${{\rm{C}}{\rm{o}}2{\rm{p}}}_{3/2}$ and −0.19 eV for ${{\rm{Co}}2{\rm{p}}}_{1/2}$, towards the lower binding energy. At the same time, the MLCO shows wider widths at half height of the main peaks than PLCO. These results demonstrate the chemical environments of LiCoO_2_ have changed by the polymer surface modification. Figure 5b shows the XPS spectrums of Co2p for PLCO and MLCO at charge state. Although their satellites look close, they are different types. Except for the chemical shifts and widths of peaks, the most important difference that mentioned before to distinguish the valence states, the ΔE values, are listed in Table 3. The ΔE of PLCO, MLCO and charged PLCO are very close, they are 15.04 eV, 15.05 eV, 15.08 eV respectively, which indicates only ${{Co}}^{3+}$ existing on the surface of them. Specially, the ΔE of PLCO and charged PLCO are very close, the valence of cobalt ions of PLCO cannot be changed by charge. However, the ΔE of charged MLCO is 15.53 eV, which falls between 15.0 eV and 16.0 eV. This fact indicates the coexistence of ${{Co}}^{2+}$ and ${{Co}}^{3+}$ on the surface of MLCO. Some ${{Co}}^{3+}$ of MLCO have been reduced to ${{Co}}^{2+}$ by charge.

**Table 2:** The Binding energy and chemical shift of 2p level for MLCO and PLCO.

	Binding energy [eV]	
	Co2p_3/2_	Co2p_1/2_
PLCO	779.95	794.99
MLCO	779.75	794.80
Chemical shift [eV]	−0.2	−0.19

**Table 3:** The Binding energy of 2p level and ΔE for MLCO and PLCO at different states.

Material	Binding energy [eV]		ΔE [eV]
	Co2p_3/2_	Co2p_1/2_	
PLCO	779.95	794.99	15.04
MLCO	779.75	794.8	15.05
Charged PLCO	779.94	795.02	15.08
Charged MLCO	780.36	795.89	15.53

For another important means to discriminate the valence states of cobalt compounds, the co-relation between the binding energies of ${{\rm{Co}}2{\rm{p}}}_{1/2}$ subshells and ${{\rm{C}}{\rm{o}}2{\rm{p}}}_{3/2}$ subshells is displayed in Fig. 5c. The binding energies of ${{\rm{Co}}2{\rm{p}}}_{1/2}$ subshells and ${{\rm{C}}{\rm{o}}2{\rm{p}}}_{3/2}$ subshells of a list of cobaltous compounds (point 1–10) and a list of cobaltic compounds (point 11–21) are given in supporting information Table S1^19^. Two different straight lines are easily fitted for the cobaltic and cobaltous compounds. The positions of the point of PLCO (cyan point “p”), and the point of charged PLCO (red point “cp”) are very close and close to the cobaltic compounds line. But there is a far distance between the point of MLCO (green point “m”) and the point of charged MLCO (blue point “cm”). The point of MLCO is almost on the line of cobaltic compounds, while the point of charged MLCO falls between the lines. This result also indicates that the coexistence of ${{Co}}^{2+}$ and ${{Co}}^{3+}$ on the surface of MLCO and only ${{Co}}^{3+}$ existing on the surface of PLCO, MLCO and charged PLCO.

The results of the XPS study indicate the polymer surface modification can change the chemical environments of LiCoO_2_, and the ${{Co}}^{3+}$ on the surface of MLCO can be reduced to ${{Co}}^{2+}$ after charge. This reduction can reduce the activity of cobalt ion on the surface of charged LiCoO_2_, then the side reactions can be retarded, as a result, the polarization can be weakened, the crystal of LiCoO_2_ can be saved and the chemical performance can be improved.

In summary, a poly N,N-bis(2-cryano-ethyl)-acrylamide coating layer on the surface of Lithium Cobalt Oxide can improve the capacity and cycle property of Lithium Cobalt Oxide battery at 4.5 V charge cut-off potential, resulting in that the capacity utilization ratio is highly improved to 80%, the relative capacity is about 220mAh g^−1^. XRD, TEM and HRTEM all have proved that the PBCEAM coating layer can protect Lithium Cobalt Oxide from crystal transformation during cycling, and the XPS analysis demonstrates that the to reduce the ${{Co}}^{3+}$ on the surface of MLCO to ${{Co}}^{2+}$ is the reason of this protection of PBCEAM. To reduce the activity of cobalt ions on the LiCoO_2_ surface plays an important role in protecting the crystal cycling at high voltage.

## Methods

### Synthesis of BCEAM

The BCEAM was synthesized by mike addition reaction of acrylamide and acrylonitrile (supporting information Figure S1a). First, 80 g acrylamide was added into 200 g dimethyl sulfoxide, 0.5 g NaOH was then added into it as catalyst after all acrylamide dissolved by stirring. Second, 125 g acrylonitrile was dropped into the solution, and stirred for another 2 hours. The temperature was controlled in the range of 30–35 °C during the whole process. The product was a slight yellowish-green clear liquid. In order to obtain pure BCEAM, the crude product was refined by silica gel H column chromatograohy with ethyl acetate - petroleum (2:1, v/v) as eluent. The pure BCEAM was finally obtained after removing the eluent by reduced pressure distillation. This pure product was confirmed using NMR (Bruker AV 300) and infrared absorption spectrum (ThermoFisher Nicolet 6700), (see supporting information Figure S1b and S1c for details).

### Coating modification of Lithium Cobalt Oxide

20 g Lithium Cobalt Oxide powders (purchased from CITIC GUOAN MGL, China, D50, 7–10 um, 1–2 ${{\rm{m}}}^{2}{{\rm{g}}}^{-1}$) and 0.4 g BCEAM were added in 20 mL acetic ether (molar concentration of BCEAM is 0.0113 ${{\rm{m}}{\rm{o}}{\rm{l}}{\rm{k}}{\rm{g}}}^{-1}$), and stirred for 2 hours at 35 °C, then filtered. The filtered Lithium Cobalt Oxide powders were dried at 30 °C for 1 hour, then poured into 100 mL 30 °C distilled water, and the aqueous redox initiators consist of 0.76 g ammonium peroxydisulfate and 0.2 g sodium hyposulfite were subsequently added into it. After 5 hours, the PBCEAM coating LiCoO_2_ (MLCO) powders were collected by pumping filtration.

### Electrochemical characterizations

For evaluation of the electrochemical performances of the MLCO, the cathode was fabricated by coating a NMP- based slurry composed of 85 wt% of MLCO, 5 wt% of PVDF binder, and 10 wt% of active carbon (super P.) on an aluminum current collector. The active material mass loading on the current collector is about 7.5 ${{\rm{m}}{\rm{g}}{\rm{c}}{\rm{m}}}^{-2}$. The half-cell (2032-type coin) was assembled by sandwiching a polypropylene separator between the MLCO cathode and a lithium metal foil. The half-cell then was activated by being filled with a liquid electrolyte of 4.35 V high voltage electrolyte (Shanshan Battery Material co. Dongguan, China). All cells were assembled in a glove box full filled with Ar.

The cycle performance was measured using a BTS-5V-20mA-type high precision battery test equipment (Neware Shenzhen, China) at the 0.2 C (0.32 ${{\rm{m}}{\rm{A}}{\rm{c}}{\rm{m}}}^{-2}$) rate under room temperature, with the cut-off potentials were set at 3.0–4.5 V.

Electrochemical impedance spectroscopy was collected by an electrochemical impedance spectrometer using a Solartron 1260 frequency response analyzer over a frequency range from 50 mHz to 100 kHz with an amplitude of 5 mV.

The cycle voltammetry (CV) was carried out using an electrochemical workstation (Arbin instrument), with metal lithium for the reference electrode and the counter electrode (scan rate 0.2 ${{\rm{mV\; s}}}^{-1}$, voltage range 3–4.5 V).

### Material characterizations

The surface morphology of LiCoO_2_ was examined using scanning electron microscopy (HITACHI SU8010). The crystal structure of LiCoO_2_ was observed by high resolution transmission electron microscope (HRTEM) using a FEI Titan G2 60–300 (AC-TEM). X-ray diffraction (XRD) date of the cathode was collected with an Empyrean (PANalytical, Holland) in the 2*θ* range from 10° to 90°. The X-ray photoelectron spectroscopy (XPS) measurements of elements on the LiCoO_2_ surface were collected with a XSAM800.

## Electronic supplementary material

Supplementary Information
