# Supplementary material for: A deep study of the protection of Lithium Cobalt Oxide with polymer surface modification at 4.5 V high voltage
Source: Sci Rep. 2018 Jan 16;8:863. doi: 10.1038/s41598-018-19176-6 (PMC5770406; doi:10.1038/s41598-018-19176-6)
Supplement: Supplementary file 1 — Supplementary Information [file 41598_2018_19176_MOESM1_ESM.pdf]

A deep study of the electrochemical performance of polymer surface modified Lithium Cobalt Oxide cathode at 4.5V high voltage

Zhixion Yang, Rengui Li, Zhenghua Deng

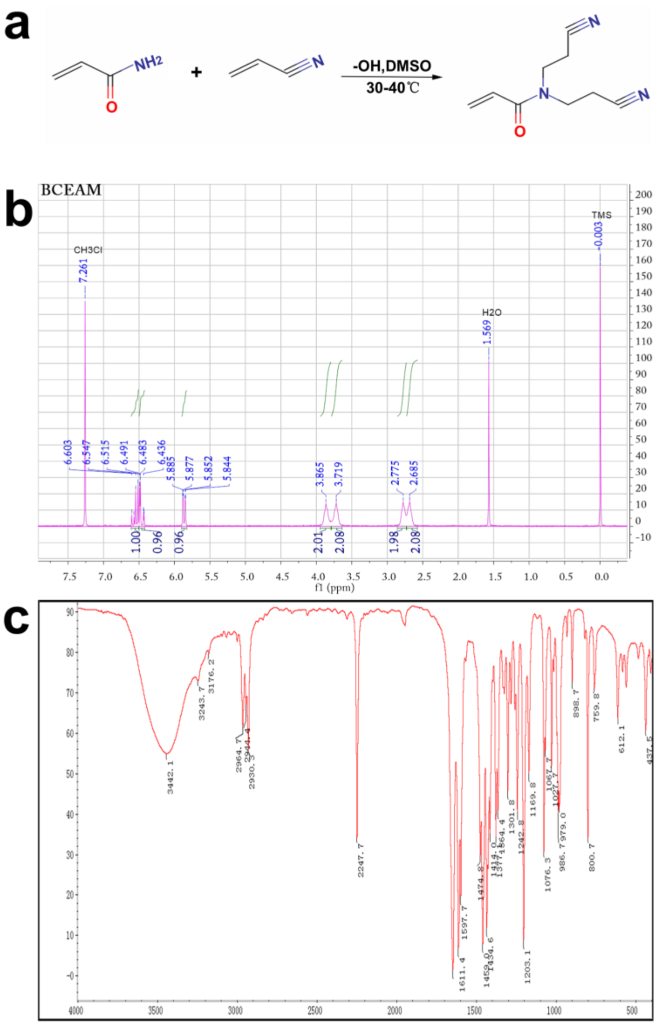

Figure S1. the reaction equation(a), nuclear magnetic resonance (NMR) spectra(b) and infrared spectra(c) of monomer N,N-bis (2-cryano-ethyl)-acrylamide (BCEAM).

NMR Dates of BCEAM:

^{1}H NMR (300 MHz, $CDCl_3$) δ 7.26 (s, 1H), 6.51 (qd, *J* = 16.6, 6.1 Hz, 1H), 5.86 (dd, *J* = 9.7, 2.4 Hz, 1H), 3.79 (d, *J* = 44.0 Hz, 2H), 2.73 (d, *J* = 27.1 Hz, 2H), 1.57 (s, 1H), 0.12 – 0.06 (m, 1H).

(1)

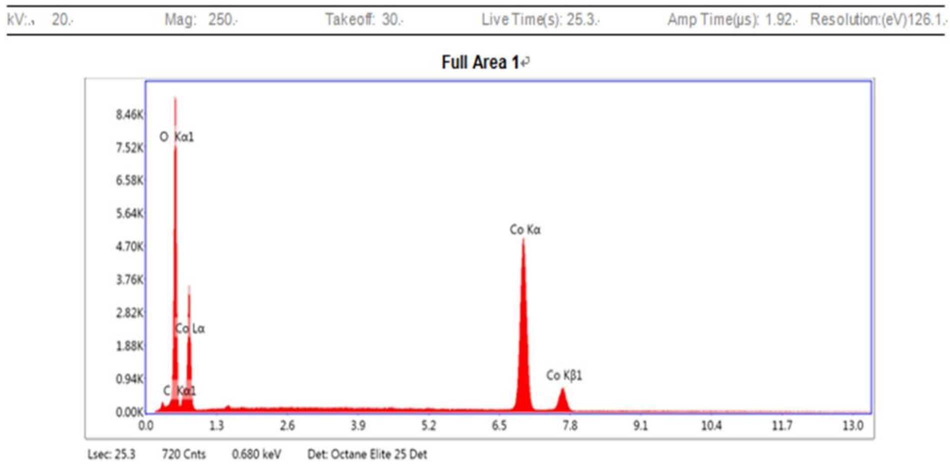

eZAF Smart Quant Results

Element	Weight %	Atomic %	Net Int.	Error %	Kratio	Z	R	A	F
C K	1.55	4.22	22.46	18.07	0.0044	1.2347	0.8750	0.2304	1.0000
O K	27.82	56.70	2098.34	6.55	0.1589	1.1873	0.8995	0.4810	1.0000
CoK	70.63	39.08	2816.53	2.08	0.6667	0.9080	1.0327	1.0076	1.0318

(2)

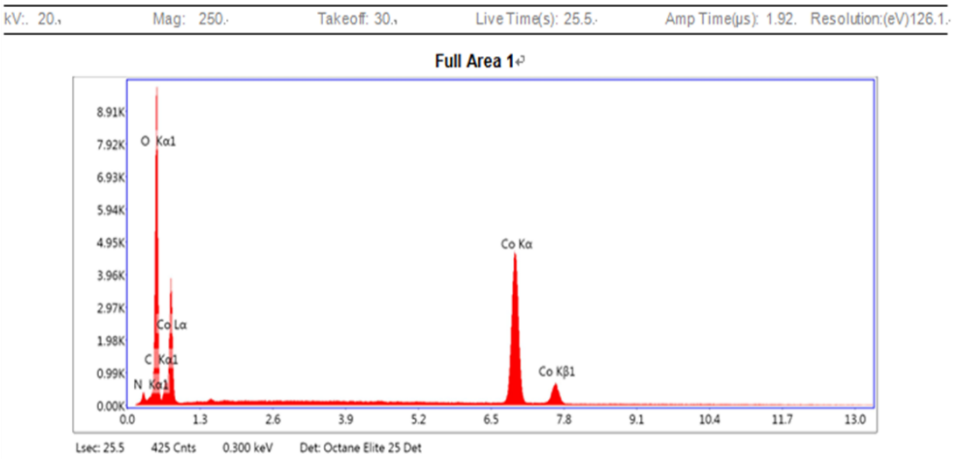

eZAF Smart Quant Results

Element	Weight %	Atomic %	Net Int.	Error %	Kratio	Z	R	A	F
C K	3.13	7.87	47.31	14.12	0.0091	1.2179	0.8826	0.2386	1.0000
N K	0.56	1.20	15.95	23.54	0.0020	1.1930	0.8955	0.2998	1.0000
O K	30.30	57.14	2200.24	6.76	0.1629	1.1709	0.9069	0.4591	1.0000
CoK	66.01	33.79	2655.52	2.10	0.6147	0.8941	1.0375	1.0088	1.0325

Figure S2. Energy dispersive X-ray analysis of pristine Lithium Cobalt Oxide (1) and modified Lithium Cobalt Oxide (2).

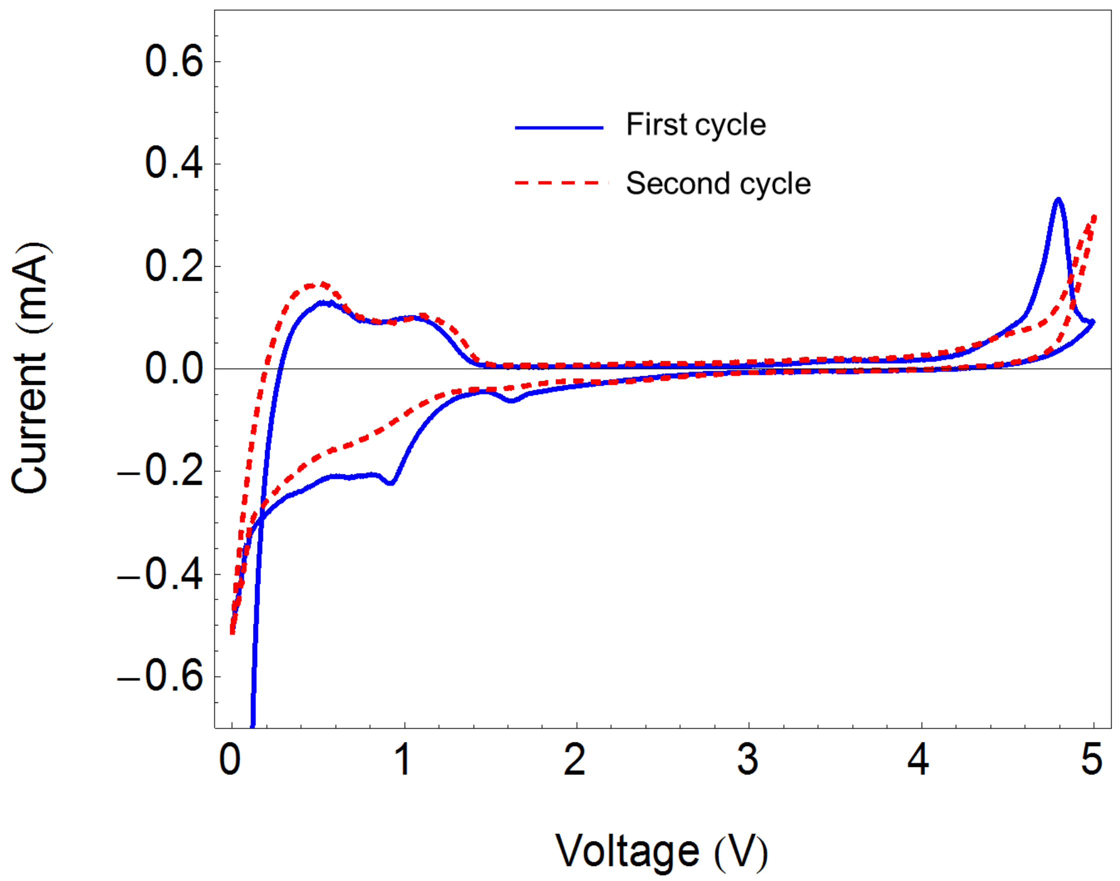

Figure S3. Cyclic voltammetry of 12663 electrolyte. The applied potential was scanned from 0V to 5V with a scan rate of 2 mVs-1 by an Arbin MSTAT 4+ system.

Table S1 The Binding Energy of Co 2p level and ΔE for cobaltous compounds (1-10) and cobaltic compounds (11-21)

	Compounds	Binding energy [eV]		ΔE [eV]
		$Co2p_{2/3}$	$Co2p_{1/2}$	
1	CoF_2	782.1	798.1	16.0
2	$Co(NO_3)_2$	781.8	797.6	15.8
3	$CoSO_4$	781.4	797.5	16.1
4	$Co(CH_3OO)_2$	781.1	797.0	16.1
5	$Co_3(PO_4)_2$	781.0	797.0	16.0

6	$\mathrm{Co}CrO_4$	780.9	796.8	15.9
7	$\mathrm{Co}Cl_2$	780.7	796.6	15.9
8	$2\ \mathrm{Co}CO_3.3\mathrm{Co}(OH)_2$	780.7	796.7	16.0
9	$\mathrm{Co}Br_2$	780.3	796.3	16.0
10	CoO	780.3	796.0	15.7
11	Co_3O_4	779.6	794.6	15.0
12	Co_2O_3	779.4	794.5	15.1
13	$K_3Co(CN)_6$	781.7	796.7	15.0
14	$[(NH_3)_5Co(O_2)Co(NH_3)_5]\ (NO_3)_4$	781.2	796.3	15.1
15	$[(NH_3)_5Co(O_2)Co(NH_3)_5]\ Cl_5$	781.5	796.5	15.0
16	$[Co(NH_3)_6]\ Cl_3$	781.7	796.7	15.0
17	$[Co(NH_3)_6]\ Br_3$	781.3	796.3	15.0
18	$[Co(NH_3)_5Br]\ Br_2$	781.0	796.1	15.1
19	$[Co(NH_3)_6]\ (NO_3)_3$	781.5	796.4	14.9
20	$[Co(en)_3]\ Cl_3$	781.2	794.9	14.7
21	$[Co(en)_3Cl_2]\ Cl$	780.0	794.9	14.9
